# Predicting population‐level impacts of projected climate heating on a temperate freshwater fish

**DOI:** 10.1111/jfb.15889

**Published:** 2024-08-28

**Authors:** Kate S. Mintram, A. Ross Brown, Samuel K. Maynard, Pernille Thorbek, Charles R. Tyler

**Affiliations:** ^1^ Department of Computer Science, College of Engineering, Design and Physical Sciences Brunel University London Uxbridge UK; ^2^ Biosciences, Faculty of Health and Life Sciences University of Exeter Exeter UK; ^3^ Global Safety Health and Environment Astrazeneca Cambridge UK; ^4^ Agricultural Solutions ‐ Ecotoxicology BASF SE, APD/EE Limburgerhof Germany

**Keywords:** agent‐based modeling, bioenergetics, IPCC, metabolic theory, three‐spined stickleback, trophic interactions

## Abstract

Climate heating has the potential to drive changes in ecosystems at multiple levels of biological organization. Temperature directly affects the inherent physiology of plants and animals, resulting in changes in rates of photosynthesis and respiration, and trophic interactions. Predicting temperature‐dependent changes in physiological and trophic processes, however, is challenging because environmental conditions and ecosystem structure vary across biogeographical regions of the globe. To realistically predict the effects of projected climate heating on wildlife populations, mechanistic tools are required to incorporate the inherent physiological effects of temperature changes, as well as the associated effects on food availability within and across comparable ecosystems. Here we applied an agent‐based bioenergetics model to explore the combined effects of projected temperature increases for 2100 (1.4, 2.7, and 4.4°C), and associated changes in prey availability, on three‐spined stickleback (*Gasterosteus aculeatus*) populations representing latitudes 50, 55, and 60°N. Our results showed a decline in population density after a simulated 1.4°C temperature increase at 50°N. In all other modeled scenarios there was an increase (inflation) in population density and biomass (per unit area) with climate heating, and this inflation increased with increasing latitude. We conclude that agent‐based bioenergetics models are valuable tools in discerning the impacts of climate change on wild fish populations, which play important roles in aquatic food webs.

## 1 INTRODUCTION

Climate change is widely considered as one of the greatest threats to ecosystems globally, with the International Panel on Climate Change (IPCC) estimating that an average temperature increase of over 1.5°C will result in 20%–30% of all species being at risk of extinction (IPCC, 2007, 2018, 2023). One third of all freshwater fish species currently face extinction, with climate change considered as a major driver (WWF, 2021). Due to climate heating, the southern boundaries of temperate climate zones in the Northern Hemisphere are moving northward by up to 0.2–0.3° of latitude per decade (Staten et al., 2018). The complexity of the ecological shifts occurring as a consequence of these climate‐driven range shifts, such as altered trophic cascades, makes it difficult to build a complete understanding of how they might play out.

Because most fish are ectotherms, meaning their physiology is directly dependent on the external environment, they are highly susceptible to climate heating. A recent report assessing future climate‐change scenarios on 11,500 riverine fish species found that in a world where the atmospheric temperature is 3.2°C warmer than present day (i.e., a scenario representing no further emission cuts after current governments' pledges for 2030), 36% of the fish species will have over half of their present‐day geographic range exposed to climatic extremes beyond their current levels and potential physiological limits (Barbarossa et al., 2021). Furthermore, the geographical ranges of many terrestrial and freshwater species have moved ~17 km poleward and 11 m up in altitude per decade in response to warming (Chen et al., 2011; IPCC, 2018). Unlike that for many marine species where there is often scope for considerable geographical movement, freshwater fish are generally enclosed within catchments, limiting their ability to extend their ranges poleward into cooler regions (Lenoir et al., 2020). Compounding this, there is growing evidence that loss of shading in riparian habitats, through widespread deforestation and other habitat alterations, is amplifying the warming of rivers across both the Northern Hemisphere and the Southern Hemisphere (Trimmel et al., 2018). A consequence of increasingly limited access to thermal refuges is that many freshwater fish will have to adapt to higher physiological temperatures or face extinction.

The Northern Hemisphere is undergoing the most rapid planetary warming, with highest temperature increases of 0.4°C/10 years predicted in the northern temperate regions, together with longer summers and shorter winters (Wang et al., 2021). Boreal regions are also predicted to undergo accelerating warming after the melting of semipermanent ice caps (Liu et al., 2020). Water temperatures in major European rivers have increased by 1–3°C over the past century (1900–2010) (European Environment Agency, 2016), whereas global mean river temperatures are projected to increase on average by 0.8–1.3°C by 2100 (relative to 1971–2000) (van Vliet et al., 2013). European freshwater fish are therefore likely to continue to experience some of the greatest net changes and variations in water temperature.

In addition to climate‐related range shifts driven by thermo‐physiological intolerance in fish, wider ecological regime shifts are likely, such as alterations in aquatic food webs. Increased water temperatures have been shown to drive large shifts in macroinvertebrate community composition in areas such as Greenland, Scandinavia, and northern Europe (Friberg et al., 2013), and as such there will likely be continuing pressure on fish to adapt their future feeding strategies and diets (Rodriguez‐Dominguez et al., 2019; Woodward et al., 2012). Riverine food webs may become more unstable, switching between bottom‐up and top‐down regulation. For example, in temperate freshwater ecosystems under climate warming, respiration rates have been shown to increase more rapidly than photosynthetic rates (Yvon‐Durocher et al., 2010, Yvon‐Durocher et al., 2012). Furthermore, increasing metabolic rates in top predators are likely to increase foraging efficiency, digestion, and growth (Hoekman, 2010). These interactions may be evaluated in experiments in which temperature and food availability are controlled, as conducted for delta smelt (*Hypomesus transpacificus*) (Fichman, 2022); alternatively, interactions may be accounted for in mechanistic models (as demonstrated here).

Most large‐scale/global studies to date have assessed the impacts of climate change on fish populations in terms of measuring and modeling species distributions and range extensions. These models are built using mathematical algorithms and have relatively low data requirements. They are therefore useful for providing crucial future projections for widescale distribution changes; however, their accuracy is limited because they often do not incorporate physiological and trophic factors. Bioenergetics models can provide mechanistic insights into temperature‐related climate change effects, provided these models also account for concurrent effects on food consumption (Railsback, 2022). Some previous studies have successfully incorporated these co‐occurring effects in agent‐based bioenergetics models for assessing the impacts of climate change on fish populations. Clark et al. (2001) and Troia et al. (2022) modeled the effects of climate change on southern Appalachian stream populations of brook trout (*Salvelinus fontinalis*) and rainbow trout (*Oncorhynchus mykiss*), and an endemic species of Guadalupe bass (*Micropterus treculii*), respectively. However, these are long‐lived species known to be sensitive to water quality and environmental change (Kovach et al., 2019; Pease et al., 2018), and the latter is a fluvial habitat specialist that is generally considered to be less able to adapt to ecological changes (Pease et al., 2018). In particular, agent‐based bioenergetics models provide valuable tools for climate change biologists because they allow investigations into changes in population dynamics from both direct and interactive effects of temperature on physiological and trophic processes. In agent‐based modeling, population dynamics emerge from complex individual‐level behaviors and interactions, meaning that the population‐level effects are not necessarily proportional to the effects modeled on individuals. Here, we present a study using an agent‐based bioenergetics model to assess the impacts of climate change on sustainability of populations across distinct ecoregions (latitudes 50, 55, and 60°N) inhabited by a generalist freshwater fish species, the three‐spined stickleback (*Gasterosteus aculeatus*).

We focus on populations of the three‐spined stickleback as a highly adaptable (generalist) fish species that is found in high abundance in freshwater and brackish‐water ecosystems throughout North America, Canada, Northern Europe, and Asia (Froese & Pauly, 2021). Sticklebacks are mesopredators, providing energy for larger fish and birds, and are thus of major importance in food webs (Gagnon et al., 2019). Excessive population growth of sticklebacks in rivers and coastal areas bordering the Baltic Sea has also been shown to result in trophic imbalances by increasing predation pressure on eggs and larvae of larger predatory fish, which are major regulators of aquatic food webs (Bergström et al., 2015).

In this study, we simulate the effects of temperature‐rise scenarios (according to IPCC predictions for 2100) for northern temperate climate zones, spanning a range of latitudes (50, 55, and 60°N) and associated levels of food availability across the Northern Hemisphere, and assess the impacts on stickleback populations. Our hypothesis is that fish (stickleback) populations will be more resilient to the effects of increasing temperatures at higher latitudes, where temperatures are currently lower and food density is higher compared to lower latitudes (Gurung et al., 2019; Huryn & Benstead, 2019).

## 2 METHODS

### 2.1 Ethics statement

Ethical approval was not sought for this study as no animals were used. Where data were used from published studies using experimental animals, the authors ensured that the care and use of those animals complied with local and/or national animal welfare laws, guidelines, and policies.

### 2.2 Model overview

**FIGURE 1:**
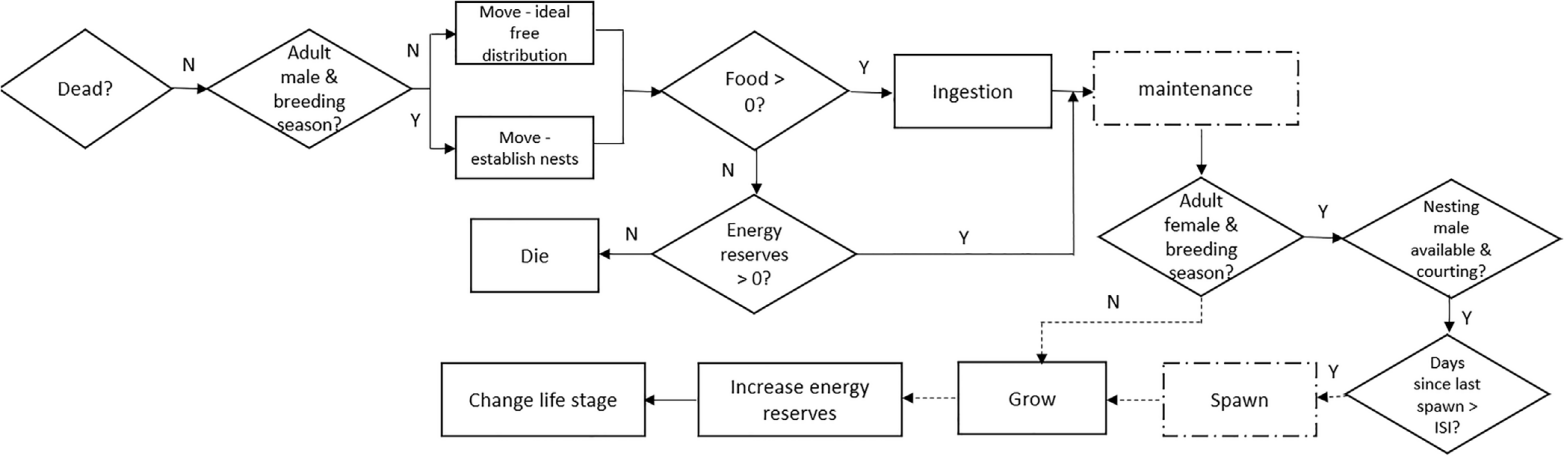
Overview of the processes undertaken by each fish (excluding eggs and larvae) every day. Dashed boxes represent processes that utilize energy reserves if assimilated energy is not sufficient to cover energetic costs. Dashed arrows represent processes that occur only if there is sufficient energy assimilated from food. Ingestion, maintenance, and growth rates scale with temperature.

A published agent‐based bioenergetics model developed for the three‐spined stickleback was adapted for this study. Full details of the model, including model parameterization, validation, and a table of equations and parameters, are documented according to the Overview, Design Concepts and Details (ODD) protocol (Grimm et al., 2020) and are presented in Appendix S1. Further details, including a full “TRAnsparent and Comprehensive model Evaludation” (TRACE) document (Grimm et al., 2014), can be found in Mintram et al. (2020). Briefly, the model simulates ingestion, assimilation, maintenance, reproduction, growth, and energy storage of each individual in the model (Figure 1). The energy allocated to metabolism, reproduction, growth, and energy storage is dependent on the total energy assimilated from food each day and the temperature of the environment. Reproduction (competition for territories and mates) and survival are additionally dependent on population density. Landscape (food density and energy content) and time (date and breeding season) variables are updated each day. The rates of physiological processes scale with temperature according to established physiological relationships. Population dynamics emerge from the interactions between individuals and their dynamic environment (food availability/competition and temperature). Stochasticity in the model is implemented at initialization (age, sex, position in the landscape) and emerges between individuals over time as assimilation (and consequently allocation) of resources is dependent on food availability and intraspecific competition.

The modeled system represents a 20‐m^2^ enclosed freshwater habitat (i.e., no migration of individuals into or out of the population) made up of 500 20 × 20‐cm patches. The environment is heterogeneous, where each patch has either a vegetated or a nonvegetated habitat type in the ratio of 1:9, with only the former containing food, amounting to 50 food patches. This setup follows wild ponds that were mapped by Whoriskey and Fitzgerald (1987) containing 10% vegetation. Individuals move across the environment according to the ideal free distribution, competing for vegetated food and nesting sites. The spatial variation in food availability drives density‐dependent competition for food across the landscape. Thus, when food is limited, the amount of food an individual ingests is dependent on both the availability of that food and the number of fish in the environment. When food is not limited, individuals ingest food according to their maximum ingestion rates.

### 2.3 Model validation

The model was validated both at the organismal and population levels. At the organismal level, modeled outputs provided accurate predictions of empirical data for body lengths (*R* ^2^ = 0.94) and wet weights (*R* ^2^ = 0.9) of fish maintained under different food rations. Additionally, ratios of egg production were 1:1.5:3.9 and 1:1.6:3.5 for observed and modeled fish maintained under low, medium, and high food rations, respectively, demonstrating accurate predictions of fecundity at different food levels.

At the population level, modeled outputs showed good predictions of long‐term annual population density and body size distributions from a UK stickleback population in spring and autumn, demonstrating that the model accurately predicts overwintering recruitment and growth. The size distribution data demonstrate that the model captures much of the variability seen in wild populations. Modeled outputs of seasonal changes in body mass and length over a year, thereby including seasonal changes in temperature and food availability, provided good predictions to two different wild populations; one was a food‐limited population, whereas the other was subject to favorable (comparatively higher food availability) environmental conditions. The full details and results of these experiments, as well as full sensitivity analysis of the control model, can be found in Mintram et al. (2020).

### 2.4 Effects of latitude on temperature and food density

The effects of projected future temperature increases were simulated on modeled stickleback populations at latitudes of 50, 55, and 60°N. The natural range of the stickleback spans between 25 and 72°N (Froese & Pauly, 2021); however, as the model is largely parameterized and validated using datasets from the United Kingdom, which spans latitudes 50–60°N, we kept our extrapolations within this temperate zone.

Modeled temperatures and food availabilities were adapted to represent the respective latitudes as follows. Monthly average temperatures were extracted using data from the GemStat database (https://gemstat.org/data/data-portal/) by calculating the average water temperatures from five sampled river sites at each respective latitude (±1°) spanning Canada, Europe, the United States, and Asia. For food availabilities, a relationship that directly quantifies primary consumer production as a function of latitude does not currently exist; thus, we have used gross primary production (GPP) as an intermediary variable. We used latitude, rather than looking at temperature alone, to capture all the ecological changes that occur with the changing geographies. GPP in rivers has been shown to increase in a linear manner with latitude (Gurung et al., 2019). The trend was quantified empirically by the authors using data collected from rivers spanning latitudes of 32–43°N in Japan and verified via a literature review of 27 studies in rivers located from 18 to 78°N across the globe. It is important to note that whereas the positive trend was significant, the error in the regression was high (*R* ^2^ = 0.16), but this is to be expected given the environmental diversity of the sites sampled. To further explore this, we generated 5000 bootstrap samples by resampling, with replacement from the raw global dataset collated by Gurung et al. (2019) to assess the robustness of the positive trend from the linear regression. The results indicated that the accuracy of the regression model is high (i.e., low bias of 0.073), whereas the precision is lower (SE of 29%). Full statistical results of the bootstrap are presented in Appendix S2. Because our simulations aim to explore trends, rather than generating absolute predictions, we considered that this dataset was suitable for parameterization. The linear relationship between GPP (mg C m^−2^ d^−1^) and latitude (°N) was calculated as follows:

GPP=38.13Lat+88.0

Primary consumer production (secondary production) (mg C m^−2^ d^−1^) in rivers was then calculated from GPP following a study by Huryn and Benstead (2019), which established a linear relationship between GPP and daily primary consumer production (*R* ^2^ = 0.64), as follows:

Primary consumer production=0.02GPP+9.04

This relationship was established using data from an Alaskan stream that has a higher latitude than those used in the simulations; however, other laboratory‐controlled and mescocosm studies have verified this general positive trend between GPP and primary consumer production (Botsch et al., 2023; Phillips et al., 2021). Daily primary consumer production (g C m^−2^) values were converted into grams per patch, where the modeled system is 20 m^−2^ and contains 500 patches, 50 of which are vegetated patches. Only vegetated patches in the model contain food. The stickleback's main food source is primary consumers, such as macroinvertebrates and zooplankton; we therefore assume that daily primary consumer production equals the available food density for the fish (US Wildlife and Fisheries, 2017). The average annual food availabilities calculated were 0.0196, 0.0211, and 0.0226 g patch^−1^ for latitudes 50, 55, and 60° N, respectively.

**FIGURE 2:**
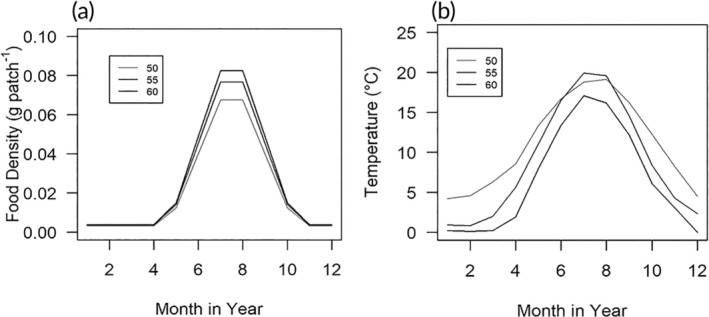
(a) Monthly food density and (b) temperature scenarios used in the model at latitudes of 50, 55, and 60°N. Legend refers to latitude (°N).

These data represent an average over the whole year, and to include seasonal variability, the model requires monthly input data. Fluctuations in monthly temperatures were modeled using the same typical seasonal trend as the data used for validation in Mintram et al. (2020). The values used in Mintram et al. (2020) were multiplied to give the same average annual food densities (g patch^−1^) calculated earlier (Figure 2). Figure 2 shows the changes in modeled temperatures and food densities over time at each latitude, with temperature decreasing and food density increasing with increasing latitude.

### 2.5 Temperature‐rise scenarios

Monthly temperatures are predicted to increase by 1.4, 2.7, and 4.4°C according to the latest IPCC long‐term scenarios (to 2100) for Shared Socioeconomic Pathways (SSPs) SSP1‐1.9, SSP2‐4.5, and SSP5‐8.5, respectively (IPCC, 2007, 2018, 2023). As increasing temperature also has a direct effect on food availability, simulated food densities also increased with the increasing temperature scenarios. In a mesocosm study that quantified macroinvertebrate biomass across a temperature gradient of 5–18°C, Scrine et al. demonstrated that the total biomass of macroinvertebrate communities (mg m^‐^^2^) increased positively with temperature (°C) (*R* ^2^ = 0.83) as follows:

$$\text{Macroinvertebrate biomass}\;\left(\log10\right)=0.138\text{Temp}+1.090$$

This was input into the model by adding 1.28, 1.46, and 1.70 to the modeled biomass (converted to log_10_ mg m‐^‐^^2^) at time point *n* for temperature‐rise scenarios 1.4, 2.7, and 4.4°C, respectively, subtracting the slope and calculating the reverse log. This calculated the additional biomass (mg m^‐^^2^), which could then be converted to biomass per patch.

### 2.6 Experimental design

The model was run for a 10‐year warm‐up period, where no results were taken, to establish a stable population, after which the temperature‐rise scenarios (0, 1.4, 2.7, and 4.4°C), and the associated changes in food density, were implemented for a further 10 years. Each 20‐year simulation was run 15 times, as this had previously been determined to generate mean and SD that are independent of run number (<5%). Thus here, population‐level effects were considered detectable if the mean density over 10 years exceeded a 5% deviation from the mean control density (Mintram et al., 2020).

Population‐level effects were quantified by comparing population density and biomass (per unit area) of reference populations (no temperature increase) with the temperature‐rise scenario on April 1 each year. This date was chosen as it represents a pre‐breeding census when the population is at its most stable (Oli & Zinner, 2003). The quantitative analyses refer to the final pre‐breeding census (year 10: April 1). Population biomass, population density, and mean metabolic rate of individuals were also analysed at the final pre‐breeding census to assess the interaction between latitude and temperature increase. These endpoints were chosen because they provide a good overview of the emergent population dynamics in the model and are most likely to be disrupted as a result of the simulated climate heating scenarios.

## 3 RESULTS

**FIGURE 3:**
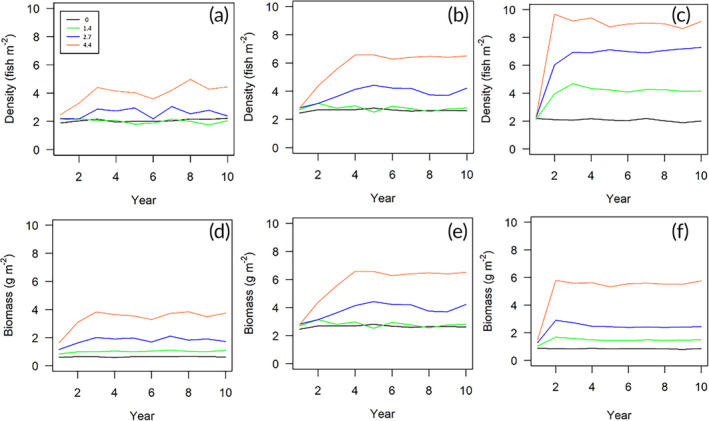
Pre‐breeding population density and biomass (m^−2^) of stickleback at latitudes of (a, d) 50°N, (b, e) 55°N, and (c, f) 60°N following IPCC (International Panel on Climate Change) temperature‐rise scenarios of 0, 1.4, 2.7, and 4.4°C. Legend refers to temperature‐rise scenario (°C). Modeled outputs represent the mean of 15 repeat model runs, output on April 1 over 10 years.

Modeled outputs demonstrated that the resilience of stickleback populations to the temperature‐rise scenarios (1.4, 2.7, and 4.4°C) generally increased with latitude. For population density and biomass, all simulated climate heating scenarios caused a temperature‐dependent increase at all simulated latitudes, except for population density following a 1.4°C increase at 50°N (Table 1; Figure 3).

**TABLE 1:** Percentage change in population density and biomass at the end of a 10‐year period in response to IPCC temperature‐rise scenarios (for 2100) of 1.4, 2.7, and 4.4°C at latitudes of 50, 55, and 60°N.

Temperature‐rise scenario (°C)	50°N		55°N		60°N	
	Density	Biomass	Density	Biomass	Density	Biomass
1.4	−8	+76	+7	+69	+108	+72
2.7	+8	+177	+61	+230	+265	+184
4.4	+100	+504	+148	+516	+358	+570

Abbreviation: IPCC, International Panel on Climate Change.

Stickleback population density was less affected by the temperature‐rise scenarios than population biomass, relative to the control (no change in temperature) scenario (Table 1). However, the positive effect of latitude on population biomass, relative to control, was evident only at the highest temperature‐rise scenario (4.4°C), suggesting the population biomass is more robust to the simulated environmental changes than population density.

The increased food availability and metabolic rates (caused by climate heating) result in a gradual increase in reproductive output (as a result of increased individual body mass and fecundity), which stabilizes at carrying capacity after 2–4 years. The population at 60°N reaches the fastest carrying capacity because the high food availability exceeds increased metabolic costs associated with growth and fecundity, with the surplus resulting in the highest reproductive output and population growth rate.

**FIGURE 4:**
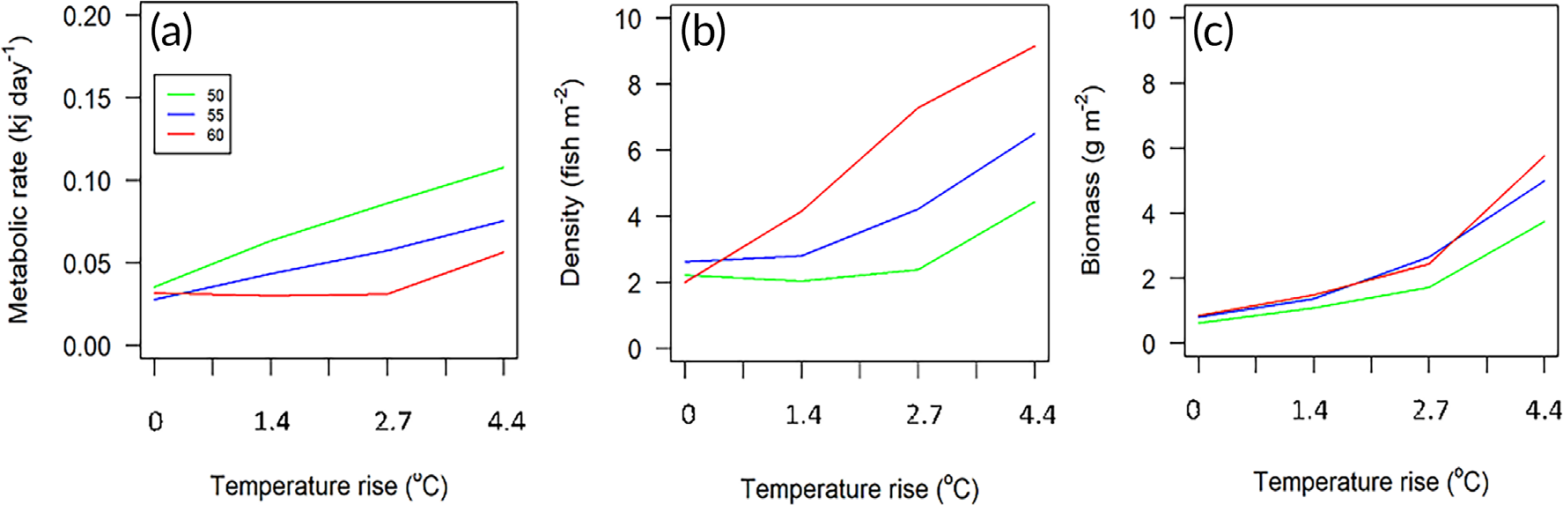
(a) Mean metabolic rate of individual sticklebacks, (b) population density, and (c) population biomass at the final time assessment point (April 1: year 10) in response to IPCC (International Panel on Climate Change) temperature‐rise scenarios (for 2100) of 0, 1.4, 2.7, and 4.4°C at latitudes of 50, 55, and 60°N. Modeled outputs represent mean values of 15 replicate runs.

The mean metabolic rate of the individual fish was lower at higher latitudes (due to lower temperatures) and increased with each temperature‐rise scenario, as expected from the implemented temperature scaling relationships (Figure 4a). The heightened metabolic rates with increasing temperature‐rise scenarios, and the concurrent increase in food availability, resulted in an increase in population density and biomass with each temperature‐rise scenario (Figure 4a,b). Population density generally increased with latitude (°N) in a northerly projection (Figure 4b), demonstrating a greater resilience to temperature increase at higher latitudes. Population biomass, on the contrary, was less affected by latitude, demonstrating only a clear effect for the temperature increase of 4.4°C (Figure 4c).

## 4 DISCUSSION

Here, we have simulated how changes in metabolic rate and energy allocation, resulting from projected climate heating‐induced changes in temperature and food availability, could affect stickleback population density and biomass across the northern temperate regions of the globe. Importantly, we show that these population‐level effects are dependent on environmental conditions (i.e., food availability and temperature) across biogeographical regions (here represented by latitudes 50–60°N). At these latitudes all the simulated climate heating scenarios for 2100, with the exception of a 1.4°C increase at 50°N, resulted in an increase in stickleback population density and biomass, and increases became more significant with increasing latitude for population density, and for population biomass at the highest simulated latitude (60°N). At this latitude the effect of climate heating on increasing population biomass exceeded proportional increases in population density. This was due to the buffering effects of density‐dependent food competition. The increased food availability associated with the temperature‐rise scenarios meant there was an increased capacity for growth of individual fish resulting in an increase in reproductive output and in turn an increase in total biomass of the population. Population abundance is more relevant to population sustainability in terms of sustaining genetic diversity (Pinsky & Palumbi, 2014). Changes in population biomass, on the contrary, are more relevant for determining effects on the food web and wider ecosystem (Delmas et al., 2017).

Metabolic theory predicts that the metabolic stress caused by reduced food availability and higher temperatures (most apparent at lower latitudes) will result in reduced energy budgets and lower quotas available for somatic growth and reproduction, which are interrelated (Wootton, 1973). This is evident in the modeled metabolic rates (Figure 4a). However, the concurrent increase in food availability with temperature increase, and with latitude, means that the increased metabolic demands for physiological maintenance can be met leaving greater energy reserves for growth and reproduction, resulting in increasingly abundant and larger fish (higher biomass). The stickleback is characterized as having high fecundity, a short life span, and high phenotypic plasticity with regard to their feeding/diet (Bretzel et al., 2021) and being relatively tolerant to pollution and changes in water quality more generally (Katsiadaki et al., 2007). Other species with a lower thermal tolerance and/or lower fecundity may have a lower capacity to increase their reproductive output and may in turn be less resilient to the climate heating scenarios simulated here. A bioenergetics model of the Guadalupe bass demonstrated an 8.7%–52.1% decrease in river reach occupancy in response to climate‐change scenarios (Troia et al., 2022). Similar to our study, the authors found that small changes in prey availability will have proportionately greater effects on growth than forecast changes in temperature. Also using a bioenergetics‐IBM, Clark et al. (2001) found that increased temperature alone resulted in increased abundances of brook and rainbow trout; however, the authors did not consider changes in prey availability.

High population abundances and biomasses of stickleback, as simulated here, can have negative consequences for aquatic food webs and ecosystems. In the Baltic Sea, interventions, such as commercial fishing, are being considered to control excessive growth of stickleback populations (BalticSea2020, 2014) to manage their negative effects, which include predating the eggs and larvae of important predatory fish species, such as perch and pike, and herbivorous fish and invertebrates, leading to eutrophication (Bergström et al., 2015). An increase in stickleback population size/biomass in higher latitudes as a result of climate heating, as demonstrated by our modeling, could therefore cause considerable trophic disruptions as described earlier.

It is also important to highlight that climate heating will also alter the behavior and/or performance of stickleback predators and prey, and these alterations may buffer or exacerbate the effects we have simulated here. For example, some fish species have shown increased levels of boldness, swimming activity, and aggression with increasing temperature (Angiulli et al., 2020; Biro et al., 2010), and this may affect prey capture ability for both the stickleback and its predators. In addition, whereas temperature‐induced changes in stickleback prey density were modeled, the potential top‐down effects of changes in stickleback predator abundance/behavior were not considered in these simulations.

When modeling food availability at a regional or global scale, it is important that data are representative of extensive river systems and spanning wide latitudes to help capture the global trends of GPP over changing latitudinal conditions. A limitation of the GPP–latitude relationship adopted from Gurung et al. (2019) is the low *R* ^2^ value provided by the model. However, as described in our methodology, bootstrapping showed the regression coefficient (i.e., the slope) to be accurate and unbiased; therefore, although there is a relatively high level of error and outliers, as would be expected from such a diverse dataset, the regression model used is considered robust for use in our stickleback model. A second limitation is that the only available study that quantified GPP with total macroinvertebrate biomass (i.e., not just one species of aquatic invertebrate) was from a stream at higher latitude than the simulations in this study (~69°N). There is therefore some uncertainty in extrapolating this for lower latitudes; however, the general positive trend has been verified at higher temperatures in the laboratory and field (Botsch et al., 2023; Phillips et al., 2021). Currently, our model is representative of a closed system and does not include migration; therefore, the results cannot be generalized to open or anadromous stickleback populations. Another limitation of our population model is the lack of consideration for potential for genetic adaptations in response to climate change. Generally, species with shorter generation times, such as the stickleback, are able to evolve and thus adapt more quickly to changes in their environment. However, adaptation is less likely for some other fish species, particularly longer‐lived species with a narrow ecological niche, including enclosed populations.

With regard to confounding environmental factors, Gurung et al. (2019) state that the uniqueness of each river in conjunction with the latitudinally related factors such as land use and land cover confounds the effects of temperatures on GPP. Furthermore, to better understand the effects of climate heating on river ecosystems, we should consider both local and latitudinal environmental conditions, including vegetation types and biomass, and anthropogenic activities in the watershed. In addition, the relationships established by Gurung et al. (2019) and Huryn and Benstead (2019) between food availability and latitude are for flowing water bodies (rivers or streams) in which environmental factors other than temperature may affect GPP. For example, GPP and ecosystem respiration are known to be affected by river water flow (Yvon‐Durocher et al., 2012) as a substantial amount of carbon may be exported downstream. In lakes and other static (lentic) water bodies, higher levels of retained GPP may provide a greater buffer for climate heating in terms of meeting the increased metabolic demands of individuals compared to flowing water bodies. Future work should include climate change–related effects on hydrology, including more frequent and extreme flooding and drought events (IPCC, 2021) and associated changes in water quality.

We conclude that our modeling results support our original hypothesis that “fish (stickleback) populations are more resilient to the effects of climate heating at higher latitudes.” To further test this hypothesis, our model would need to be adapted and validated for stickleback populations at lower latitudes, including open, migrating populations, and also for other fish species with differing life histories to the stickleback. Nevertheless, the results of our current simulations are in keeping with the body of literature, demonstrating the importance of trophic interactions to population‐level responses to climate change.

## AUTHOR CONTRIBUTIONS

This manuscript was written with contributions from all authors. All authors approved the final version of the manuscript. All authors contributed to the design of the experiments. Kate S. Mintram performed the experiments and analysed the data.

## FUNDING INFORMATION

The author(s) received no financial support for the research, authorship, and/or publication of this article.

## CONFLICT OF INTEREST STATEMENT

The employment affiliation of the authors is shown on the cover page. The authors prepared the manuscript during the normal course of their employment. This paper is the exclusive professional work product of the authors. None of the authors has appeared in any litigation or regulatory proceedings during the past 5 years related to the contents of this paper.

## Supporting information


**Appendix S1.** Supporting information.

**Appendix S2.** Supporting information.

## Data Availability

The NetLogo model and associated input files are available as a supplement to this study.
