# Supplementary material for: Predicting population‐level impacts of projected climate heating on a temperate freshwater fish
Source: J Fish Biol. 2024 Aug 28;105(6):1715–23. doi: 10.1111/jfb.15889 (PMC11650920; doi:10.1111/jfb.15889)
Supplement: Supplementary file 1 — Appendix S1. Supporting information. [file JFB-105-1715-s001.docx]

**Appendix 1: An Overview, Design and Details (ODD) document supporting:**

***Predicting population-level impacts of projected climate heating on a temperate freshwater fish***

Kate S. Mintram, A. Ross Brown, Samuel K. Maynard, Pernille Thorbek, Charles R. Tyler.

**Purpose**

The purpose of the model is to simulate realistic population dynamics of the three-spined stickleback, including responses to spatial and temporal variation in environmental conditions, to investigate population level effects of environmental stressors

**Entities, state variables and scales**

The entities in the model are the spatial units (comprising the landscape) and individual fish with their own energy budget. The model environment represents a 2 dimensional 20 m^2^ pond comprising of 500 20 L patches measuring 20cm (length) * 20 cm (width) * 50cm (depth). Individuals are distinguished into one of four life stages: eggs, larvae, juveniles or adults. All sticklebacks are characterised by the state variables age, body weight (g), body length (cm). Juveniles and adults are further characterised by energy reserves (kj), sex (male or female) and location. Energy reserves are implicit for eggs and larvae as their life-cycle processes are not dependent on food availability. Adult males possess the state variable breeding status: Boolean; if they are able to establish territories then they are nesting and exhibit nesting behaviour, territory size (m^2^) and courtship success probability. Adult females have an inter-spawning interval (days between spawnings) which determines the frequency of spawning events, and a batch size (eggs per spawning event). Spatial units are characterised by water volume (L), food density (g) and food quality (kj/ g). The overall environment is characterised by temperature and season.

**Process overview and scheduling**

Each individual in the model possesses its own energy budget. Each of the following processes (highlighted in bold) will occur over each time step in sequential order. Entities are processed in a random sequence and individual fish update their state variables each day.

**Update time and landscape:** Temperature and season are updated. Food density and energy content of each patch is replenished at the start of each day.

**Survival**: An individual’s daily mortality rate is determined by four main factors: developmental mortality (eggs only), density dependent egg cannibalism (eggs only), starvation (juveniles and adults), and background mortality (including predation; all life-stages).

**Movement:** Foraging individuals (juveniles and adults) move across patches according to the ideal free distribution (Milinski, 1979, 1984) in order to maximise ingestion rates. Sexually mature adult males move to find nesting sites in the breeding season, and adult females move to find a breeding partner.

**Ingestion and energy intake:** Juveniles and adults assimilate a fixed proportion of energy dependent on the maximum ingestion rate of the individual (determined by food availability), the energy content of the food, and the number of individuals on the patch. The food density of the patch depletes accordingly each day. Eggs are ingested throughout the breeding season by all eligible fish in the population, including the parent male.

**Maintenance:** Juveniles and adults pay maintenance costs from assimilated energy or energy reserves according to their active metabolic rate, which is increased for breeding males to account for the costs of breeding behaviours. Individuals die if the costs of maintenance are greater than the sum of assimilated energy and energy reserves. Mass and temperature have scaling effects on metabolic rate.

**Reproduction:** Fish reproduce during the breeding season. Females allocate energy to reproduction prior to growth if there is sufficient assimilated energy remaining after maintenance costs have been paid. Males establish territories and implicitly undertake courtship behaviours. All male reproductive parameters are set prior to maintenance so that the costs of breeding can be paid for in ‘*maintenance*‘. Male courtship behaviours and territory quality are criteria that females use to choose where they deposit their eggs.

**Growth:** Larvae grow at a constant rate. Juveniles and adults undergo somatic growth if assimilated energy is available after the costs of maintenance, and reproduction for adult females, have been paid. If there is insufficient energy to achieve maximum growth, growth rates are adjusted accordingly. This sub-model determines total length (cm) and structural mass (g).

**Energy reserves:** Juveniles and adult males allocate energy to reserves once the costs of maintenance and somatic growth have been paid. Energy is stored in the form of lipids; the total energy content of the reserves (kJ) is converted into mass (g), which is added onto the structural mass of the individual. When food is limited reserves are used to cover maintenance and reproduction costs until reserves are depleted. Fluctuations in energy reserves result in fluctuations in total body mass.

**Change life-stage:** Eggs develop into larvae and larvae into exogenously feeding juveniles once they are fully developed at the end of a temperature dependent incubation period. Juveniles develop into adults during the breeding season once they reach the length at which sexual maturity is onset.

**Design concepts**

*Basic principles:* Individual energy budgets follow fundamental principles of physiological ecology (Sibly and Calow, 1986) and scale with body mass and temperature according to known allometric laws (Sibly et al., 2013). Food availability and temperature drive seasonal discrepancies in the allocation of available energy to key life-history processes and thus regulate population dynamics.

*Emergence:* Landscape heterogeneity emerges as a result of individuals depleting food reserves over a single day. Population dynamics (age and size structure, spatial distribution and abundance of fish) emerge as food density and quality results in differential energy allocation among individuals at different life stages.

*Adaptation*: Several behaviours in the model are implicitly adaptive. Reproductive behaviour is the primary adaptive trait driving reproductive success as male traits (nest building, courtship, territory size, and nest quality) determine female choice. Juvenile and adult movement is implicitly adaptive as individuals disperse themselves based on patch profitability to maximise energy gain. Adult males search for optimal breeding grounds to establish territories which reduce mortality risk and increase the chance that a female will spawn in his nest.

*Sensing*: Stickleback sense the presence of conspecifics in the local (territorial males and spawning females) and global (all adults and juveniles) environment. Adults sense the presence of eggs and/or larvae in the nest and adjust their territorial behaviour accordingly. Juveniles and adults sense patch conditions (food density/ quality) and adjust their movement (move to find a higher quality patch) and behaviour (e.g. establish territory, spawn) accordingly.

*Interaction:* Direct interaction occurs during breeding where a paired male and female carry out both communicative signalling (courtship) and physical contact (fertilisation). These interactions are modelled implicitly. Each male will continue interacting with its fertilised eggs and larvae until the larvae leave the nest. Indirect interaction occurs via competition for food and territory sites and mates during reproduction.

*Stochasticity:* Habitat types are distributed randomly within the system. At initialisation, age, position, and sex are the results of stochastic processes.

*Observation*: Total abundance, number of individuals at each life stage, and population biomass (g) will be recorded daily.

*Input data:* Water temperature and food density data is read from an external file*.* Water temperature recordings were taken throughout the year at a lake in Slapton Ley, Devon, at a depth of 0.5 m (Turner et al., 2013). Food density trends follow trends in water temperature (described later in this section).

*Initialisation*: The model begins on January 1^st^ with only juveniles present within the system. An initial density of 100 juveniles was chosen as it is within the range of stickleback found in the wild (see section 2, *calibration*) but low enough to maximise model speed. Age is drawn from a uniform distribution (i.e. set randomly) between 142 and 232 days post hatch (dph) based on a three month breeding season between May and July. Length (cm) is set according to age (Eq. 5*b*) and weight (g) is allometric to body length (Eq. 6). The position of individuals within the pond is allocated based on a preference for complex vegetated habitat, as displayed in wild populations (Candolin and Selin, 2012). Juveniles are randomly allocated to a vegetated patch; as densities have been observed at up to 60 fish m^-2^ in the wild (Whoriskey and FitzGerald, 1985), there is assumed to be no exclusion of juveniles from vegetated patches outside of the breeding season. Energy reserves are set to 50% of their maximum to represent declined reserves in winter.

The pond patches are setup with 10% vegetation cover following a wild pond which was mapped by Whoriskey and Fitzgerald (1987). All of these vegetated patches are considered potential territories for males during the breeding season. The remaining patches are set as open water, all of which are set as potential breeding-grounds; however this can be changed by the user. All vegetative patches have an energy content set randomly between 17 and 25 kj g^-1^ (Wootton, 1994). Food density and temperature is read from the external file.

**Sub-models**

**Table 1.** Equations used in each sub-model of the IBM with parameter descriptions and units. Parameter values and sources can be found in table 2.

| **Sub-model** | **Equation** | **Parameter descriptions** |
| --- | --- | --- |
| **Survival** | **Eq 1. Natural mortality**  $M_{m}=M_{u}M^{b}$ | M_w_: Natural mortality probability at mass M  M_u_: Natural mortality probability at unit mass (1 g)  M: mass (g)  b: Allometric scaling factor |
|  | **Eq 2. Egg cannibalism**  $EC= EC_{a}D+EC_{b}$ | EC: Egg cannibalism probability  EC_a_ : EC constant  D : Global adult and juvenile (fish length >= 1.5 cm) density (fish m^-2^)  EC_b_ : EC intercept |
| **Ingestion** | **Eq 3. Ingestion rate**  ${IG= IG}_{max}e^{\frac{-E}{k}(\frac{1}{T}-\frac{1}{T_{ref}})} \frac{X}{(h+X)} L^{2}$ | IG: Ingestion rate (g cm^-2^ day^-1^)  IG*_max_*: maximum ingestion rate ( g cm^-2^ day^-1^)  X: Food density (g l^-1^)  h: half-saturation coefficient (g l^-1^)  L: Body length (cm)  E: Activation energy (eV)  k: Boltzmann’s constant (eV K^-1^)  T: Temperature (K) |
| **Maintenance** | **Eq 4. Metabolic rate**${MR= B}_{0}M^{\frac{3}{4}}e^{(-\frac{E}{kT})}$ | MR : Metabolic rate  B_0_: taxon-specific normalisation constant  M: Mass (g)  E: Activation energy (eV)  k: Boltzmann’s constant (eV K^-1^)  T: Temperature (K) |
| **Growth** | **Eq 5. Growth rate**  $GR=(Ke^{\frac{-E}{k}\left( \frac{1}{T}-\frac{1}{T_{ref}} \right)}) (L_{m}-L)/3$ | GR: Growth rate (cm day^-1^)  K: Growth constant (cm day^-1^)  L_m_: Asymptotic length (cm)  L: Length (cm)  E: Activation energy (eV)  k: Boltzmann’s constant (eV K^-1^)  T: Temperature (K) |
|  | **Eq 6. Length: mass conversion**  $M=m_{a}L^{m_{b}}$ | M: Mass (g)  m_a_: mass constant  m_b_: mass exponent |
| **Reproduction** | **Eq 7. Territory size**  If D > 20 fish m^-2^  TS = 0.063  If D < 1.3 fish m^-2^  TS = 0.54  If 20 > adult male density > 1.3 fish m^-2^    $TS={T_{a}D}^{T_{b}}$ | TS : Territory size (m^2^)  T_a_ : TS constant  D: Male density (fish m^-2^)  T_b_ : TS exponent |
|  | **Eq 8. Courtship success**  $CS=C_{a}Ln\left( TS \right)+C_{b}$ | CS : Courtship success probability  C_a_ : CS constant  TS : Territory size (m^2^)  C_b_ : CS intercept |
|  | **Eq 9. Reproduction rate**  ${R= r}_{m}m$ | R: Maximum reproduction rate (kj day^-1^)  r_m_: maximum reproduction rate per unit mass (kj g^-1^ day^-1^)  M: Mass (g) |

*Update environment and landscape*

Date and season are updated every time step. Temperature (K) and food density (g) are read from an external file and are updated every 5 days. Temperature data was taken from a lake in Devon, UK (Turner et al., 2013) at a depth of 0.5 m which matches the depth of the modelled system. This data was interpreted from a graph which reported monthly mean temperatures. Food density data follows the patterns of temperature, such that food increases with increasing temperature, but the minimum and maximum food densities were chosen based on the available literature (see section 2). The energy content of each vegetated patch changes daily and is set randomly between 17 and 25 kj g^-1^ (Wootton 1994) to maintain a heterogeneous environment. Open water patches have a food density of 0.

*Survival*

Egg mortality occurs in the form of developmental mortality (all natural mortality caused by mutations, incorrect egg development and infections), egg cannibalism, and predation. Laboratory data on developmental mortality from the literature and from a study undertaken by the authors in the absence of predation showed a mean hatching success of 92%. There is no evidence that hatching success is affected by temperature (within ranges which eggs would be subject to in the wild) or incubation time, as displayed in the parameterisation data (Hagen, 1967; Candolin et al., 2008; personal observations). To accommodate this, we set a constant daily mortality rate (*N_e_*) of 0.014 for the first 6 days of incubation (the minimum incubation time in the model). This results in a constant overall rate of developmental egg mortality independent of development time.

Stickleback eggs are subject to high levels of cannibalism which can vary in intensity between populations (Foster 1988, Ostlund-Nilsson et al., 2006). In the wild, eggs in the nest are predated by raiding males and females (hetero-cannibalism) and by the guarding male parent (filial cannibalism) (Pitcher, 1986). Whoriskey and FitzGerald (1985) quantified density dependent cannibalism in the stickleback in a semi-wild study and this data was used in the model (Table 1, Eq. 2). The study did not explicitly account for resource availability; however, a study by Candolin (2000) demonstrated that low rations did not significantly increase cannibalism in the stickleback. Similarly, Klug et al., (2006) found that male condition in the sand goby, a species with a similar breeding strategy to the stickleback, did not affect the rate of egg cannibalism. There is therefore little empirical evidence for the energy-based hypothesis for egg cannibalism presented by Rohwer (1978), whereby cannibalism is presented as a strategy to increase body condition when food supplies are low. We therefore do not explicitly relate egg cannibalism to food density in the model; rather, we relate cannibalism to overall fish density as recorded by Whoriskey and FitzGerald (1985). Cannibalistic fish ingest eggs once the food density of the patch is depleted. Egg predation by other species is assumed to be minimal because of the paternal guarding investment undertaken by the male stickleback. It is therefore assumed in the model that non-cannibalistic egg predation only occurs when the guarding male dies.

Background mortality (including predation) affects larvae, juveniles and adults. Since there are no data quantifying mortality rates for larval stickleback in the wild, this parameter was calibrated (see *calibration* for details). Daily mortality rates (*M_l_*) are considered to stay constant for the duration of the larval stage to account for low motility potential and consequent high rates of predation. For juveniles and adults, background mortality is set according to the natural mortality at unit mass equation (Table 1, Eq. 1) where an increase in body mass results in a reduced daily mortality rate (Lorenzen, 1996). This equation incorporates all forms of mortality including predation, background mortality and starvation, and follows the general theory that as fish get older, and larger, mortality decreases (Wootton, 2002). Since starvation is already included in the model, the M_u_ parameter, which was reported as 0.00781 by Lorenzen et al (1996) for temperate pond fish (when converted from annual to daily mortality), was reduced and calibrated (see section 2, *calibration* for details).

Juveniles and adults are subject to starvation if the sum of assimilated energy and energy reserves does not cover maintenance costs (see *maintenance*).

*Movement*

Eggs and larvae do not move. Foraging stickleback (juveniles and adults outside of a breeding cycle) follow the ideal free distribution as described by Milinski (1979) and Milinski (1984) where individuals distribute themselves between food patches in the ratio of the patch profitability (Pitcher 1986). Once sexually mature, adult males move to find nesting sites and adult females move to find receptive breeding partners (see *Reproduction*).

During the breeding season, movement is adapted so that non-cannibalistic fish continue to follow the ideal-free distribution as described above, but cannibalistic fish are excluded from adult male territories as they pose a threat to offspring in the nest. Thus, if there are no unowned patches (i.e. patches which are not within a males territory) which follow the ideal free criteria, cannibalistic fish move to any owned patch with a food density > 0. If there are no unowned patches with a food density > 0, cannibalistic fish move to any unowned patch.

*Ingestion and energy intake*

Individual ingestion rates follow a type II functional response and are dependent on temperature and food density (Table 1, Eq. 3). The model was parameterised from data which quantified attack rate as a function of food density (Volsett and Bailey, 2011) and ingestion rate as a function of attack rate (Heller and Millinksi, 1979). If the density of food on the patch is insufficient to meet the maximum ingestion rates of all the individuals on that patch, each individual acquires an equal amount of food proportional to their body mass.

Ingestion rates of cannibalistic individuals in the breeding season are additionally dependent on the number of eggs in the system. Cannibalistic fish are defined in the model as individuals with a body length >= 1.5cm, since mouth gape size prevents smaller fish from cannibalising eggs. There are no studies quantifying mouth gape size in stickleback, so a body length >= 1.5 cm was considered to be a sensible estimate, based on observations in the laboratory (personal observation), to represent individuals capable of cannibalism. After ingesting all of the available food on the patch, cannibalistic fish ingest eggs until they have reached their maximum ingestion rate or until there are no more eggs available. Individuals do not explicitly move to acquire eggs; rather, the density of eggs available for ingestion is spread evenly throughout the population of cannibalistic fish and the additional energy is added to each individual energy budget. Since the proportion of filial cannibalism and nest raiding by other individuals is unknown (Pitcher, 1986) and varies between populations (Foster 1988, Ostlund-Nilsson et al., 2006), this seems the most effective way to model egg ingestion.

Sticklebacks are omnivorous but the diet is generally dominated by two prey categories: zooplankton and the larvae and pupae of chironomids (Wootton, 1974). The energy content of food (*E_x_*) was taken as 17 – 25 kj g^-1^ dry weight (Wootton, 1974) and an assimilation efficiency (*A_e_*) of 0.95 was taken from Cui (1987). Since there are no reported assimilation efficiencies for the stickleback, the assimilation used here refers to the common minnow, *Phoxinus phoxinus.*  Eggs have an energy content equal to E_c_. The total energy assimilated in a day is thus equal to: ingestion rate * ((*E_x_* + *E_c_)* * *A_e_*).

*Maintenance*

In the model we assume that eggs and larvae have sufficient energy in the yolk sac to cover maintenance costs and thus maintenance is not explicitly modelled for these life stages. Maintenance costs in juveniles and adults follow the active metabolic rate (AMR, basal metabolism + the energy costs of swimming), below which the organism cannot survive (Table 1, Eq. 4). Meakins (1975) recorded the routine rate of spontaneously active stickleback to be approximately twice that of their basal metabolic rate (BMR), which is consistent with general theory (Fry, 1947; Tytler and Calow, 1985). It is assumed that the daily costs of swimming in a static water body remain relatively constant from day to day because the fish do not migrate and do not need to swim against flow rates. To account for active metabolism, we therefore apply a multiplication factor of 2 to the costs of BMR (see section 2).

Over the course of the breeding season, the costs of maintenance in adult males are significantly increased as individuals undergo a complex series of movement-based behaviours (Chellappa et al., 1989). These behaviours consist of establishing a territory, nest building, courtship displays, and the guarding and fanning of young. The period between territory establishment and larvae fleeing the nest is defined as a breeding cycle and typically lasts 14 days. Meakins and Walkey (1975) (recalculated by (Smith, 1991)) estimated the costs of active metabolism during a breeding cycle to be 4.3 times greater than the costs of BMR. This multiplication factor is applied to the costs of BMR.

Energy reserves are utilised in juveniles and adults in instances where the energy assimilated does not cover maintenance costs. Once all of the energy reserves are used up, the individual dies (Sibly et al., 2013).

Growth

Eggs do not grow. Fish length (total length, cm) is used as the primary element of growth in the model. Larvae grow at a constant rate of 0.033 cm/ day at 15 °C (pers. obvs). Larvae always grow at their maximum rate because they rely on their yolk sac for energy. Juveniles and adults increase in body length according to the von Bertalanffy growth function (Table 1, Eq. 5) which results in decreasing growth rates as fish get larger. The model was parameterised from laboratory fish (n = 12) reared at 15°C for the first 60 days of life (personal observations) and using data from Froese and Pauly (2016). Larvae growth rates are dependent on temperature, whereas juvenile and adult growth rates are dependent on temperature and food availability. Thus, as fish density increases and resources become limiting, growth rates of juveniles and adults decrease. Body length is converted to mass according to the allometric equation (Table 1, Eq. 6), parameterised from 29,975 sticklebacks (Froese and Pauly, 2016). The energy costs of synthesising new tissue are calculated as the daily addition of somatic body mass, taking into account the costs of synthesising new tissue$(E_{c}+E_{s})$. In juveniles and adults, the allometric equation here is multiplied by 1/1.3 to ensure that only the costs of somatic growth are accounted for (see details below). If less energy is available than required for maximum growth rates, a lower rate is calculated for the energy available.

The average condition factor (CF, an index of the extent to which the total weight of a fish is high for its length, calculated as mass / length^3^ x 10^6^) of stickleback in the wild is 1.3 (Chellappa et al., 1995) and it is therefore assumed that body mass calculated from the allometric equation (Table 1, Eq. 6) refers to fish with a CF of 1.3. Juvenile and adult mass fluctuates accordingly depending on individual energy reserves, whereby individuals with maximum energy reserves represent the highest possible mass, and individuals with no energy reserves refers only to the structural mass of the individual. The condition factor for the structural mass of the fish, or the somatic condition factor, is 1 (Chellappa, et al., 1995). This sub-model accounts for the structural mass of the individual only, and body mass is therefore calculated as ${(M}_{a}L^{M_{b}}) \frac{1}{1.3}$. The mass of energy reserves are added onto the structural mass in ‘*energy reserves’*. Since CF in larvae is assumed to be constant and independent of food availability, the mass calculated from the allometric equation refers to total mass for larvae.

Reproduction

This process is executed by adult stickleback in the breeding season (1^st^ May to 30^th^ June). Adults are sexually mature once they have reached a total length of 4.5 cm (Froese and Pauly, 2016; Paepke, 1984).

***Males***

Each day, males which have reached sexual maturity acquire nests on available vegetated patches and establish territories around these patches according to their territory size (Table. 1, Eq. 6). If there are no vegetated patches available, males will establish territories around open water patches. Thus, a male may establish a nest on a single vegetated patch, but the territory surrounding this nest may be a mixture of vegetated and open water patches. This is consistent with evidence from wild stickleback demonstrating that individuals show a preference for concealed nest sites over open water nest sites (Black, 1971; Moodie, 1972; Kynard, 1978; Hagen, 1967; Sargent and Gebler, 1980; Krakk et al., 2000). Territories are established on a first come first serve basis so that competition for territories is random and independent of size. Although there is some evidence that larger males display a competitive advantage over smaller males when choosing territory sites (Rowland, 1989; Dufresne et al., 1990; Kraak et al., 2000), we did not implement size-dependent territory acquisition into this sub-model because it significantly slowed down the IBM without causing any changes in overall outputs. Since male sticklebacks ferociously guard their territories, it is presumed that once a male has established a nest, he cannot be excluded from that nest. A male which acquires a territory is defined as a nesting male. When searching for territories, non-nesting males search for un-owned patches within a radius that is set to be slightly larger than his allocated territory size. This ensures that patches are allocated to an individual’s territory in a near continuous manner surrounding the turtle, as is likely to occur in the wild. The chances that a male will successfully court a female is set by his courtship success probability (Table 1, Eq. 7), which is dependent on territory size.

The breeding season lasts 3 months (90 days) during which a single male can complete as many breeding cycles as possible until he is outcompeted or dies (Kynard, 1978; Wootton, 1984). Each breeding cycle lasts 14 days and consists of a nest building phase (day 0); a courtship phase (days 1 - 4) which determines if a female will spawn; and a parental phase (nest guarding and fanning of embryos and fry: days 2 - 14) (Wootton, 1984; Kynard, 1978; van de Assam, 1967). A male can acquire a maximum of 5 clutches in days 1 - 4 of each breeding cycle; after which point he will reject any more females into his territory (van de Assam; 1967; Wootton, 1984). If a male fails to acquire a clutch within this period, the cycle is set back to 1 (courtship phase). This is to avoid males guarding a territory for a full breeding cycle without being able to acquire any egg clutches after day 4. The owner will abandon his territory at day 14 of his cycle and begin searching for another space as previously described (Kynard et al., 1978). A male can acquire a final clutch in an already established territory on the last day of the season (day 90) resulting in these males completing their final cycle into August (up to day 104). In wild populations, the time spent in parental care of fry is often variable and can range from abandonment prior to the development of free-swimming fry (van de Assam, 1967; Kynard, 1978), to continuing the parental phase until the fry leave the nest independently (Kynard, 1978). In the model, this is dependent upon which day of the breeding cycle the clutch was acquired. It is assumed that if a male is removed from his territory through mortality, all of the eggs in his nest will die as they are unable to survive without aeration or guarding from predators.

Males do not fast during the breeding season, but they rarely leave their territory during a breeding cycle in order to maximise the survival of their young (Wootton 1984). In the model, males undergoing a breeding cycle can only forage on the patch that they occupy (i.e. the nest patch), and can forage as usual outside of a cycle.

***Females***

Once the breeding season commences, temperature and photoperiod cues result in adult females which have reached a given length to become sexually mature and begin spawning (McPhail, 1977; Baggerman, 1958). During each inter-spawning interval (ISI), energy is accumulated for expenditure on egg production and at the end of the ISI an egg batch is deposited if there is a receptive male available. A female’s ISI is a function of body mass whereby larger females have a shorter ISI (Wootton, 1974). Females with a weight <= 0.49 have an ISI of 9, females with a weight >= 0.94 have an ISI of 3 and females between these weights have an increasing ISI with weight between 3 and 9 days (Brown-Peterson and Heins, 2009; Wootton et al., 1995). These ISI’s were defined based on an empirical study undertaken by Wootton (1973). The maximum rate of energy allocation to egg production per day increases linearly with mass (Table 1, Eq. 9). The number of eggs produced from the accumulated energy is calculated as the total energy accumulated within an ISI / M_h_ (E_c_ + E_s_). Females produce eggs up to a maximum number per egg batch, which is calculated as the maximum rate of energy allocation to egg production per day * ISI. This prevents females continuously producing eggs if there are no receptive males. An average fertilisation rate of 0.935 is used for all females, as the literature indicates no changes in fertilisation rate with age or size (Frommen et al., 2008; Barber and Arnott, 2000).

Females first search for an available nest in a vegetated patch (Kraak et al., 1999); if none are available she will search for an open water nest. In the wild, although only one female can visit a nest at a given time, more than one female can visit each day (van den Assem, 1967; Wootton, 1984). In the model, two females can visit a nest at one given time to account for the one day time step implemented. An available territory is therefore defined as containing one male guarding no more than 4 clutches in days 1 – 4 of his breeding cycle, with no more than 1 female already present. The probability that she spawns in the nest of the owner of that territory is determined by the courtship success probability of the male owner.

When energy accumulated from ingested foods does not meet the requirements of reproduction between successive spawnings, the cost of egg production is subsidised from available energy reserves (Wootton 1977, Wootton 1994). In the lab, females will continue to produce eggs even when the food supply is not sufficient to maintain their body weight (Wootton 1977, Wootton and Evans 1976), suggesting that reproduction will continue to be prioritised until energy reserves run out. This is also supported in wild populations, where most individuals lose mass and decrease in somatic condition (Crivelli and Britton 1987, Wootton et al., 1978) and energy reserves are depleted throughout the breeding season (Wootton, et al., 1978).

Energy reserves

Energy is mainly stored as lipids (Chellappa, et al., 1989) costing 54 kj g^-1^for synthesis and storage and yielding 39.3 kj g^-1^ (Jobling 1994, Schmidt-Nielsen 1997), giving a cost of synthesis of 14.7 kj g^-1^. According to Chellappa (1995), the maximum condition factor (see *growth*) reported in wild fish was 1.4 compared to a somatic condition factor of 1. Energy reserves in the model are thus stored up to a maximum threshold proportional to 40% of an individual’s structural mass. For simplicity, we assume that all energy is stored as lipid because glycogen accounts only for a very small proportion of total energy reserves (Chellappa et al., 1989; Chellappa, 1995). The mass of energy reserves is added onto the structural mass of the individual to give a total mass.

Energy reserves in eggs and larvae are implicit as we assume that there is sufficient energy in the yolk sac to cover maintenance, and maximum growth costs in larvae.

Change life-stage

Eggs develop into endogenously feeding larvae following a temperature-dependent incubation period of 11 days at 15°C (pers obvs). Larvae develop into exogenously feeding juveniles following a temperature-dependent incubation period of 4 days at 18.5 degrees (Swarup, 1958). Juveniles develop into sexually mature adults once they reach 4.5 cm (Froese and Pauly, 2016; Paepke, 1984).

| **Table 2.** Default parameter values of stickleback PEB model with sources. All fish related weights refer to wet weights, whereas food density parameters refer to dry weights. | | | | |
| --- | --- | --- | --- | --- |
| **Symbol & Definition** | | **Value** | **Unit** | **Reference** |
| *E* | Activation energy | 0.457 | eV | Killen et al. (2010) |
| *A_e_* | Assimilation efficiency | 0.95 |  | Cui (1987) |
| *E_x_* | Energy content of food | Unif (17, 25) | kJ g^-1^ | Wootton (1994) |
| *B_o_*  *IG_max_*  *h* | Taxon-specific normalization constant  Maximum ingestion rate  Half saturation coefficient | 7.8 x 10^6^  0.006947  0.0000367 | kJ g^-1^ day^-1^  g cm^-2^ day^-1^  g l^-1^ | calculated from Meakins (1975)  Volsett and Bailey (2013) |
| Ec | Energy content of tissue Energy content lipid | 7 39 | kj g^-1^  kj g^-1^ | Peters (1983) Jobling (1994); Schmidt-Nielsen (1997) |
| *E_s_* | Energy cost of synthesising tissue  Energy cost of synthesis lipid | 3.6    14.7 | kJ g ^-1^    kJ g ^-1^ | calculated from Sibly and Calow (1986, pp. 54-5)  Jobling (1994); Schmidt-Nielsen (1997) |
| *L_m_* | Maximum asymptotic length | 6.7 | cm | Froese and Pauly (2016) |
| *K*  *L_h_*  *M_h_*  *L_p_* | Growth constant  Length at hatch  Mass at hatch  Length at sexual maturity | 0.0193  0.45  0.00156  4.5 | cm day^-1^  cm  g  cm | pers.obvs  pers.obvs  Wootton (1973)  Paepke (1984) |
| *M_a_*  *M_b_* | mass constant  mass exponent | 0.0068  3.28 | ---  --- | Froese and Pauly (2016)  Froese and Pauly (2016) |
| *M_u_*  *b*  *M_l_*  *EC_a_*  *EC_b_*  N_e_ | Natural mortality probability at unit mass  Allometric scaling factor  Larval background mortality rate  Egg cannibalism constant  Egg cannibalism intercept  Egg natural mortality rate | 0.0051  -0.427  0.26  0.0049  -0.0133  0.014 |  | Lorenzen (1996) and calibrated  Lorenzen (1996)  calibrated  Whoriskey and FitzGerald (1985)  Hagen (1967); Candolin et al. (2008); pers. obvs |
| rm    f  ISI*_a_*  ISI_b_  T*_a_* T*_b_*  C*_a_*  C*_b_* | Maximum rate of energy allocation to reproduction  Fertilisation rate  inter-spawning interval constant  inter-spawning interval intercept  Territory size constant  Territory size exponent  Courtship constant  Courtship intercept | 0.74    0.935  -13.215  15.444  0.653  -0.797  0.0577  0.895 | kj g^-1^ day^-1^ | Hagen (1967)    Frommen et al. (2008); Barber and Arnott (2000) Wootton (1974)  Wootton (1974)  van de Assam (1967)  van de Assam (1967) |
| *Tref* | Reference background temp | 288.15 | kelvin |  |
|  |  |  |  |  |

**References**

Baggerman, B., 1958. An experimental study on the timing of breeding and migration in the three-spined stickleback. Arch Neerl Zool. 12(2):105-317.

Barber, I., Arnott, S.A., 2000. Split-clutch IVF: a technique to examine indirect fitness consequences of mate preferences in sticklebacks. Behaviour 137(7):1129-1140.

Black, R., 1971. Hatching success in the three-spined stickleback (*Gasterosteus aculeatus*) in relation to changes in behaviour during the parental phase. Anita. Behav. 19(3):532-541.

Brown-Peterson, N.J., Heins, D.C., 2009. Interspawning interval of wild female three‐spined stickleback Gasterosteus aculeatus in Alaska. J Fish Biol. 74(10):2299-2312.

Candolin, U., 2000. Changes in expression and honesty of sexual signalling over the reproductive lifetime of sticklebacks. Proc R Soc Lond B Biol Sci. 267(1460):2425-2430.

Candolin, U., Engström‐Öst, J., Salesto, T., 2008. Human‐induced eutrophication enhances reproductive success through effects on parenting ability in sticklebacks. Oikos. 117(3):459-465.

Candolin, U., Selin, M., 2012. Density-Dependent Habitat Selection in a Growing Threespine Stickleback Population. ‎Int J Zool.

Chellappa, S., Huntingford, F., Strang, R., Thomson, R., 1989. Annual variation in energy reserves in male three‐spined stickleback, *Gasterosteus aculeatus* L.(Pisces, Gasterosteidae). J Fish Biol. 35(2):275-286.

Chellappa, S., Huntingford, F., Strang, R., Thomson, R., 1995. Condition factor and hepatosomatic index as estimates of energy status in male three‐spined stickleback. J Fish Biol. 47(5):775-787.

Crivelli, A.J., Britton RH. 1987. Life history adaptations of *Gasterosteus aculeatus* in a Mediterranean wetland. Environ Biol Fishes. 18(2):109-125.

Cui, Y., 1987. Bioenergetics and growth of a teleost *Phoxinus phoxinus* (Cyprinidae). Ph.D. thesis, University of Wales.

Foster, S.A., 1988. Diversionary displays of paternal stickleback. Behav Ecol Sociobiol. 22(5):335-340.

Froese, R., Pauly, D., 2016. FishBase.World Wide Web electronic publication. Available at: www.fishbase.org, version (01/2016). Accessed on 01.06.16.

Frommen, J.G., Luz, C., Mazzi, D., Bakker, T.C., 2008. Inbreeding depression affects fertilization success and survival but not breeding coloration in threespine sticklebacks. Behaviour, 145(4):425-441.

Fry, F., 1947. Effects of the environment on animal activity. Publ. Ontario Ii ‘ish. Rcs. Lab. 68.‘. Univ Toronto.

Gillooly, J.F., Brown, J.H., West, G.B., Savage, V.M., Charnov, E.L., 2001. Effects of size and temperature on metabolic rate. Science, 293:2248–2251.

Hagen, D.W., 1967. Isolating mechanisms in threespine sticklebacks (*Gasterosteus*). J Fish Res Board Can. 24(8):1637-1692.

Jobling, M., 1994. Fish Bioenergetics. London, UK: Chapman and Hall.

Killen, S.S., Atkinson, D., Glazier, D.S., 2010. The intraspecific scaling of metabolic rate with body mass in fishes depends on lifestyle and temperature. Ecol Lett. 13(2):184-193.

Klug, H., Lindström, K., Mary, C.M.S., 2006. Parents benefit from eating offspring: density-dependent egg survivorship compensates for filial cannibalism. Evolution. 60(10):2087-2095.

Kraak, S.B., Bakker, T.C., Hočevar, S., 2000. Stickleback males, especially large and red ones, are more likely to nest concealed in macrophytes. Behaviour, 137(7):907-919.

Kynard, B.E., 1978. Breeding behavior of a lacustrine population of threespine sticklebacks (*Gasterosteus aculeatu*s L.). Behaviour 67(3):178-206.

Lorenzen, K., 1996. The relationship between body weight and natural mortality in juvenile and adult fish: a comparison of natural ecosystems and aquaculture. J Fish Biol. 49(4):627-642.

McGurk, M.D., 1986. Natural mortality of marine pelagic fish eggs and larvae: role of spatial patchiness. Mar Ecol Prog Ser. 34(3):227-242.

McPhail, J.D., 1977. Inherited interpopulation differences in size at first reproduction in threespine stickleback, *Gasterosteus aculeatus* L. Heredity 38(1):53-60.

Meakins, R., Walkey, M., 1975. The effects of parasitism by the plerocercoid of *Schistocephalus solidus* Muller 1776 (Pseudophyllidea) on the respiration of the three‐spined stickleback *Gasterosteus aculeatus* L. J Fish Biol. 7(6):817-824.

Meakins, R.H., 1975. The effects of activity and season on the respiration of the three-spined stickleback, *Gasterosteus aculeatus* L. Comp Biochem Physiol A Physiol. 51(1):155-157.

Milinski, M., 1979. An evolutionarily stable feeding strategy in sticklebacks1. Zeitschrift für Tierpsychologie. 51(1):36-40.

Milinski, M., 1984. Competitive resource sharing: an experimental test of a learning rule for ESSs. Anim Behav. 32(1):233-242.

Moodie, G.E.E., 1972. Morphology, life history, and ecology of an unusual stickleback (Gasterosteus aculeatus) in the Queen Charlotte Islands, Canada. Can J Zool. 50(6):721-732.

Ostlund-Nilsson, S., Mayer, I., Huntingford, F.A., 2006. Biology of the three-spined stickleback. CRC Press.

Paepke, H.J., 1983. Die Stichlinge., volume 10 of Die Neue Brehm-Buecherei. A. Ziemsen Verlag DDR, WittenBerg, Germany.

Paul, S. S., Michael, J. D., 1994. International study of Artemia IX. Lipid level, energy content and fatty acid composition of the cysts and newly hatched nauplii from five geographical strains of Artemia. Mediterranea Artemia training course and site survey. University of Rhode Island.

Peters, R.H., 1983. The Ecological Implications of Body Size. Cambridge University Press, Cambridge.

Pitcher, T.J., 1986. Functions of shoaling behaviour in teleosts. In: The behaviour of teleost fishes. Springer.

Rohwer, S., 1978. Parent cannibalism of offspring and egg raiding as a courtship strategy. Am Nat. 112 (984):429-440.

Sargent, R.C., Gebler, J.B., 1980. Effects of nest site concealment on hatching success, reproductive success, and paternal behavior of the threespine stickleback, *Gasterosteus aculeatus.* Behav Ecol Sociobiol. 7(2):137-142.

Schmidt-Nielsen, K., 1997. Animal physiology: adaptation and environment. Cambridge University Press.

Sibly, R.M., Calow, P., 1986. Physiological ecology of animals. Blackwell Scientific Publications.

Sibly, R.M., Grimm, V., Martin, B.T., Johnston, A.S., Kułakowska, K., Topping, C.J., Calow, P., Nabe‐Nielsen, J., Thorbek, P., DeAngelis, D.L., 2013. Representing the acquisition and use of energy by individuals in agent‐based models of animal populations. Methods Ecol Evol. 4(2):151-161.

Smith, C., 1991. Filial cannibalism as a reproductive strategy in care-giving teleosts? Neth J Zool. 42(4):607-613.

Swarup, H., 1958. Stages in the development of the stickleback *Gasterosteus aculeatus* (L.). Development. 6(3):373-383.

Turner, S.D., Rose, N.L., Goldsmith, B., Harrad, S., Davidson, T.A., 2013. OPAL Water Centre monitoring report 2008–2012. London: OPAL.

Tytler, P., Calow, P., 1985. Fish energetics: new perspectives.

van den Assem, J., 1967. Territory in the three-spined stickleback Gasterosteus aculeatus L.: an experimental study in intra-specific competition. Brill.Archive.

Whoriskey, F.G., FitzGerald, G.J., 1985. Sex, cannibalism and sticklebacks. Behav Ecol Sociobiol. 18:15-18.

Whoriskey, F.G., FitzGerald, G.J., 1987. Intraspecific competition in sticklebacks (Gasterosteidae: Pisces): does mother nature concur?. ‎J Animal Ecol. 939-947.

Wootton, R.J., 1973. The effect of size of food ration on egg production in the female three‐spined stickleback, *Gasterosteus aculeatus* L. J Fish Biol. 5 (1): 89-96.

Wootton, R.J., 1974. The inter-spawning interval of the female three-spined stickleback, *Gasterosteus aculeatus.* J Zool. 172(3):331-342.

Wootton, R.J., Evans, G., 1976. Cost of egg production in the three‐spined stickleback (*Gasterosteus aculeatus* L.). J Fish Biol. 8(5):385-395.

Wootton, R.J., 1977. Effect of food limitation during the breeding season on the size, body components and egg production of female sticklebacks (Gasterosteus aculeatus). J Anim Ecol. 823-834.

Wootton, R.J., Evans, G., Mills, L., 1978. Annual cycle in female three‐spined sticklebacks (*Gasterosteus aculeatus* L.) from an upland and lowland population. J Fish Biol. 12(4):331-343.

Wootton, R.J., 1994. Energy allocation in the threespine stickleback. The evolutionary biology of the threespine stickleback. 114-143.

Wootton, R.J., 1984. A functional biology of sticklebacks. Univ of California Press.

Wootton, R.J., Fletcher, D.A., Smith, C., Whoriskey, F.G., 1995. A review of reproductive rates in sticklebacks in relation to parental expenditure and operational sex ratios. Behaviour. 132(13):915-933.

Wootton, R.J., Smith, C., 2000. A long-term study of a short-lived fish: the demography of *Gasterosteus aculeatus*. Behaviour. 137(7):981-997.

Wootton, R.J., 2002. Ecology of teleost fishes. 3rd Ed, London. Chapman and Hall.

Wootton, R.J., Adams, C.E., Attrill, M.J., 2005. Empirical modelling of the population dynamics of a small population of the threespine stickleback, *Gasterosteus aculeatus*. Environ. Biol. Fishes 74(2):151-161.

Wootton, R.J., 2007. Over‐wintering growth and losses in a small population of the threespine stickleback, *Gasterosteus aculeatus* (L.), in mid‐Wales. Ecol Freshwat Fish. 16(4):476-481.
