# Supplementary material for: Predicting population‐level impacts of projected climate heating on a temperate freshwater fish
Source: J Fish Biol. 2024 Aug 28;105(6):1715–23. doi: 10.1111/jfb.15889 (PMC11650920; doi:10.1111/jfb.15889)
Supplement: Supplementary file 2 — Appendix S2. Supporting information. [file JFB-105-1715-s002.docx]

**Appendix 2.**

We generated 5000 bootstrap samples by resampling with replacement from the global data set collated by Gurung et al. (2019). For each bootstrap, we calculated the linear regression coefficient to assess the robustness of the positive trend. Bootstrapping assigns measures of accuracy to sample estimates. The bias quantifies the difference between the observed statistic and the bootstrap value, and indicates the accuracy of the data. The standard error is an estimate of the standard deviation of the sampling distribution of the mean, and it indicates the precision of the observed data. The point estimate is a single value computed from the sample data to serve as the "best guess", or estimate, for the linear regression coefficient.

The data showed a small bias of 0.073 with a standard error of 10.997 on a point estimate of 38.13. The former indicates that the accuracy of the regression model is high and unbiased, but the precision is low , as indicated by the high standard error (~29% of the point estimate).

Point estimate


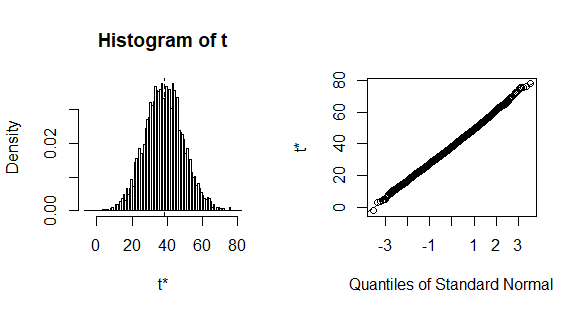


Figure 1. Histogram showing a normal distribution around the point estimate.
